# Amiloride derivatives enhance insulin release in pancreatic islets from diabetic mice

**DOI:** 10.1186/1472-6823-5-9

**Published:** 2005-12-08

**Authors:** Subhadra C Gunawardana, W Steven Head, David W Piston

**Affiliations:** 1 Department of Molecular Physiology and Biophysics, 702 Light Hall, Vanderbilt University, Nashville, TN 37232, USA

## Abstract

**Background:**

Amiloride derivatives, commonly used for their diuretic and antihypertensive properties, can also cause a sustained but reversible decrease of intracellular pH (pH_i_). Using dimethyl amiloride (DMA) on normal rodent pancreatic islets, we previously demonstrated the critical influence of islet pH_i _on insulin secretion. Nutrient-stimulated insulin secretion (NSIS) requires a specific pH_i_-range, and is dramatically enhanced by forced intracellular acidification with DMA. Furthermore, DMA can enable certain non-secretagogues to stimulate insulin secretion, and induce time-dependent potentiation (TDP) of insulin release in mouse islets where this function is normally absent. The present study was performed to determine whether pH_i_-manipulation could correct the secretory defect in islets isolated from mice with type 2 diabetes.

**Methods:**

Using two mouse models of type 2 diabetes, we compared a) pHi-regulation, and b) NSIS with and without treatment with amiloride derivatives, in islets isolated from diabetic mice and wild type mice.

**Results:**

A majority of the islets from the diabetic mice showed a slightly elevated basal pH_i _and/or poor recovery from acid/base load. DMA treatment produced a significant increase of NSIS in islets from the diabetic models. DMA also enabled glucose to induce TDP in the islets from diabetic mice, albeit to a lesser degree than in normal islets.

**Conclusion:**

Islets from diabetic mice show some mis-regulation of intracellular pH, and their secretory capacity is consistently enhanced by DMA/amiloride. Thus, amiloride derivatives show promise as potential therapeutic agents for type 2 diabetes.

## Background

As is widely known, nutrient-stimulated insulin secretory response in the pancreatic β cell consists of three distinct components. These include: a) an initial peak (first phase) triggered by Ca^2+^, b) augmentation of the Ca^2+^-triggered response (second phase), and c) a memory that persists after removal of the nutrient, causing enhancement of subsequent secretory responses (time-dependent potentiation) [1-4]. The first phase of the insulin response is initiated by ATP derived from glucose metabolism, which leads to membrane depolarization through closure of ATP-dependent K^+ ^channels (K_ATP _channels) and consequent entry of extracellular Ca^2+ ^through voltage-gated Ca^2+ ^channels. This influx of Ca^2+ ^triggers the release of a small pool of secretory granules, thus producing the initial peak of the insulin response [1]. This peak is followed by a sustained second phase of insulin release that lasts through the duration of exposure to the nutrient, mediated through augmentation of the Ca^2+^-triggered first-phase response [1]. Time-dependent potentiation (TDP), a positive memory induced during this acute response, magnifies subsequent secretory responses to all secretagogues [2,4-6].

Islet intracellular pH (pH_i_) exerts a strong influence on all aspects of the insulin secretory response, as well as other related functions such as Ca^2+ ^influx and K^+ ^retention [7-17]. These functions are enhanced by a decrease of pH_i_, and inhibited by an increase of pH_i_. Our recent work on isolated mouse islets has demonstrated that NSIS requires a specific pH_i_-range, and is greatly enhanced by decreasing islet pH_i _using DMA [7]. The basal pH_i _in mouse islets ranges from 6.9 to 7.2. Treatment with DMA, an inhibitor of the Na^+^/H^+ ^exchanger, brings islet pH_i _to the lower range of 6.4–6.8. This decrease of pH_i _in turn causes a dramatic enhancement in insulin secretion induced by metabolic secretagogues, and enables certain non-secretagogues to stimulate insulin release [7]. Another remarkable effect of DMA is to enable glucose to induce TDP, a function normally absent in mouse islets [7]. Hence, DMA has a consistently favorable effect on all aspects of the insulin response in isolated mouse islets. Previous studies have shown similarly positive effects of amiloride derivatives on insulin secretion in other species as well [8,12-14,16].

Thus, it is possible that: a) a mis-regulation of intracellular pH may contribute to the defect in insulin secretion found in type 2 diabetes; and b) Forcing the islet pH_i _to a lower range may correct/improve this secretory defect. To test these hypotheses, we monitored insulin secretion and islet pH_i _in two mouse models of type 2 diabetes. Regulation of islet pH_i _and the influence of amiloride derivatives on insulin secretion and TDP in isolated islets from diabetic mice were compared to similar parameters in islets from wild type mice.

Results from both models of type 2 diabetes demonstrate some abnormalities in pH_i_-regulation, and consistently favorable effects of DMA/amiloride on both direct insulin secretion and TDP in isolated islets. These results suggest the potential value of amiloride derivatives in treatment of type 2 diabetes

## Methods

### Animals

Mouse models of type 2 diabetes i.e. strains NON/LtJ (Diabetic NON mice; stock number 002423) and KK/Upj-A<y>/J (Diabetic KK mice; stock number 002468) were purchased from Jackson Laboratories, Bar Harbor, ME. C57BL6 mice (Harlan Sprague Dawley, Indianapolis, IN) were used as wild type controls. All animals were males, and the control mice were 7–9 weeks of age. The diabetic KK and NON mice were used at 9–20 weeks and 5–9 months, respectively, in order to allow time for NIDDM to develop. The body weight range was 20–30 g for the control mice, and 40–80 g for the diabetic mice. The animals were fed standard laboratory chow, and cared for according to the guidelines of the Vanderbilt Institutional Animal Care and Use Committee.

### Media

Pancreatic islets were isolated in Hanks Balanced Salt solution, and HEPES-buffered Krebs Ringer Bicarbonate solution (KRBH) was used for the static incubations in secretion experiments. The components of KRBH are as follows: 128.8 mM NaCl; 4.8 mM KCl; 1.2 mM KH_2_PO_4_; 1.2 mM MgSO_4_; 2.5 mM CaCl_2_; 5 mM NaHCO_3_^-^; 10 mM HEPES; and 0.1% bovine serum albumin. The medium pH was maintained at 7.4. Basal KRBH used for pre-incubation and non-stimulated controls contained 2.8 mM glucose, while the stimulating media contained either 16.7 mM glucose, or other secretagogues as indicated in the presence of 2.8 mM glucose. The non-glucose secretagogues included 20 mM alpha-ketoisocaproate (αKIC), or 20 mM 2-amino-bicyclo[2,2,1]heptane-2-carboxylic acid (BCH). αKIC, a mitochondrially-metabolized secretagogue, is a metabolic product of the amino acid leucine. BCH is a non-metabolizable analog of leucine, and stimulates insulin secretion presumably through allosteric activation of mitochondrial dehydrogenases. For forced decrease of islet pH_i_, 40 μM DMA was added to the medium to produce intracellular acidification. In preparation for imaging experiments, islets were cultured in RPMI 1640 culture medium supplemented with 10% heat-inactivated fetal bovine serum, 100 U/ml penicillin, 0.1 g/L streptomycin and 11 mM glucose.

### Isolation of islets

A modified version of the collagenase digestion method described by Lacy and Kostianovsky [18] was used. Mice were terminally anesthetized with intra-peritoneal injection of Ketamine/Xylazine (80/20 mg/Kg). Pancreas was removed, placed in ice-cold Hanks solution and minced with scissors. Collagenase (3 mg/ml) was added and the mixture shaken in a 37°C water bath until the tissue was adequately digested. The mixture was then centrifuged, supernatant removed and the pellet re-suspended in Hanks solution. Centrifugation and re-suspension were repeated several times to remove exocrine tissue. The final pellet was re-suspended either in basal KRBH medium for secretion experiments or RPMI medium for islet culture. Islets were hand picked under a stereomicroscope.

### Culture of islets

The method described by Arkhammar et al. 1998 [19] was used with minor modifications [7,20,21]. 35 mm culture dishes with glass-bottomed wells (Mat-Tek corporation) were used. The dishes were pre-prepared by coating the wells with human extracellular matrix (BD Biosciences). Freshly isolated islets were placed carefully in each well, covered with RPMI medium containing 11 mM glucose, and cultured at 37°C in 95% O_2 _and 5% CO_2_. Under these conditions, the cells in the islet spread out within 14 days, greatly reducing the islet thickness and making it particularly suitable for imaging with confocal microscopy. Islets cultured under these conditions exhibit normal responses of Ca^2+^, NAD(P)H and insulin release to glucose stimulation, as described in previous studies [19-21] and confirmed in our preliminary experiments.

### Intracellular pH measurements

As described in our previous studies [7], islet pH_i _was monitored by confocal microscopy, using carboxy-seminaphthorhodofluor-5 (SNARF5) [22], a pH-sensitive fluorescent indicator. Prior to imaging, cultured islets were maintained in RPMI medium containing 5 mM glucose for at least 48 hours. On the day of the experiment, RPMI medium was removed, and islets were washed and placed in basal KRBH medium. SNARF5-AM (5 μM final concentration) was added and incubated for one hour at 37°C. Loaded islets were placed on a warmed stage in a humidified and temperature-controlled chamber at 37°C, and monitored with a F-Fluar 40 × 1.3 NA oil immersion lens of a LSM510 confocal laser-scanning microscope (Zeiss). Islets were excited at 514 nm with an argon laser, and the emission fluorescence was collected in the band-widths 568–589 nm and 621–643 nm (peak emission at 580 and 630 nm) using the Meta detector (Zeiss). Time-series images (2.56 μs/pixel) were collected for 5–20 minutes as was suitable for each experiment. A stable baseline was obtained before the actual recording for each experimental condition started. LSM software was used to calculate the ratio between the two emission fluorescence values from selected well-loaded regions in each islet. This ratio was proportional to the islet pH_i_. The results were analyzed using LSM software, Graphpad Prism, and Microsoft Excel. One representative recording for each experimental condition is shown in the results section, and the value n denotes the number of recordings done with different islets for each condition. A standard curve was prepared by fixing the islet pH_i _at known values (ranging from 5.5 to 9, with 5–10 islets for each pH), using a KRBH medium containing 100 mM K^+ ^and 20 mM nigericin to equilibrate the pH inside and outside cells.

### Secretion measurements

All incubations were done in a 37°C water bath. Freshly isolated islets were pre-incubated for one hour in basal KRBH containing 2.8 mM glucose. Islets were then divided into groups and stimulated with different compounds as indicated in the results section, for one hour. Control group was maintained in basal glucose. Each group consisted of 4–5 tubes containing 4 islets per tube. At the end of the stimulation period, samples were collected for insulin measurement by radio-immuno-assay (performed by the DRTC Core facility at Vanderbilt University). Islet insulin content was measured after freezing islets overnight in 1% Triton-X. Insulin secretion was expressed as fractional release, i.e. the percentage of total insulin content released over the period of stimulation. In the experiments monitoring TDP, after pre-incubation islets were exposed to high (16.7 mM) glucose with or without intracellular acidification for 40 minutes, while the control group was exposed to basal glucose. Subsequently all groups were rested in basal glucose for 20 minutes, and stimulated with high glucose for 40 minutes prior to collection of samples for insulin assay. The value n denotes the number of times each experiment was repeated using islets from different mice.

### *In vivo *treatment

Effect of oral treatment with amiloride derivatives on insulin secretion from isolated islets was monitored in diabetic NON mice. Mice were divided into three groups, i.e. DMA-treated; amiloride treated; and untreated control. DMA (estimated dose: 1 mg/Kg/day) or amiloride (estimated dose: 5 mg/Kg/day) was administered in drinking water to the test groups for one week. At the end of the week mice were euthanized and insulin secretion was measured from isolated islets as described earlier.

### Statistical Analysis

Values are expressed as mean ± SEM. Groups were compared using paired Student's *t *test. In secretion studies, n denotes the number of times each experiment was repeated with islets from different mice. In imaging experiments for pH_i_, n denotes the number of islets imaged for each condition.

## Results and Discussion

The influence of islet pH_i _on insulin secretion has been reported in a number of studies [7-17]. Our recent work in isolated mouse islets has demonstrated the critical dependence of NSIS on an appropriate pH_i_, and the ability of pH_i_-lowering agents to dramatically enhance all components of the insulin response [7]. Type 2 diabetes is characterized by a progressive decline in insulin secretion in the β cell in addition to peripheral insulin resistance. Since islet pH_i _plays a critical role in NSIS in normal islets, it is likely that a mis-regulation of pH_i _may contribute to this secretory defect, which could be corrected through manipulation of islet pH_i_. Thus, the present study was aimed at exploring the beneficial effects of pH_i_-lowering drugs on insulin release from isolated islets in type 2 diabetes.

We used two mouse models of type 2 diabetes obtained from Jackson laboratories: the KK/Upj-A<y>/J mice (Diabetic KK mice) and the NON/LtJ mice (Diabetic NON mice). Diabetic KK mice are a congenic strain developed as a model for obesity and type 2 diabetes. They develop obesity, hyperglycemia (300–500 mg/dl), insulin resistance and hyperinsulinemia (10 ng/ml) by 8 weeks of age [23]. Diabetic NON mice are an inbred strain, originally developed as a control strain for the well-known IDDM model of NOD/LtJ. NON mice are genetically related closely to NOD mice but have a diabetes resistant MHC haplotype *H2*^*nb*1 ^(K^b^, A^nb1^, E^k^, D^b^), and are homozygous for the retinal degeneration allele *Pde6b*^*rd*1^. NON mice have genes predisposing to type 2 diabetes, evidenced by early impaired glucose tolerance and development of maturity onset obesity and hypoinsulinemia. At 20 weeks of age NON males exceed 40 g in body weight, with blood glucose and insulin levels reported around 200 mg/dl and 1 ng/ml, respectively [24].

**Figure 1:**
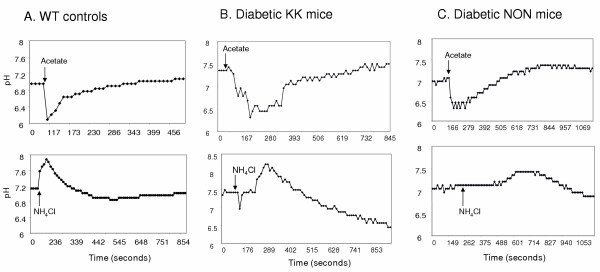
**Islets from diabetic mice show abnormalities in pH_i _regulation: **Cultured islets loaded with SNARF5-AM were excited at 514 nm, and emission fluorescence was collected at 580 and 630 nm. Islet pH_i _was calculated from the ratio of emission fluorescence. Acetate (40 mM) or NH_4_Cl (5 mM) was added where indicated. One representative recording for each condition is shown. 10–20 islets were imaged for each condition. A. Wild type islets: Basal pH_i _range 6.9–7.1. Fast recovery from drastic pH_i_-change produced by acid or base load. B. Islets from diabetic KK mice: Basal pH_i _range 7.3–7.5. Recovery from acid load is slower. Recovery from base load is followed by overcompensation. C. Islets from diabetic NON mice: Basal pH_i _similar to wild type controls. Response to base load is slower. Recovery is from acid/base load is followed by overcompensation.

Basal intracellular pH in wild type (WT) mouse islets ranges from 6.9 to 7.2. The strong buffering mechanisms in the β cells ensure quick recovery from the pH_i_-changes induced by treatment with a weak acid or weak base (acid/base load) [7,8], and Fig. 1A]. Approximately 70% of the islets isolated from diabetic mice showed abnormalities in their pH_i_-regulation. In diabetic KK mice, the basal islet pH was higher than that in WT mice (Fig. 1B). Islets isolated from both diabetic strains showed slower recovery from acid/base load, sometimes followed by overcompensation (Fig. 1:B&C). Thus, type 2 diabetes is associated with some mis-regulation of islet pH_i_. Regardless of whether these abnormalities play a role in insulin secretion, treatment with amiloride derivatives is likely to improve NSIS in diabetes.

**Figure 2:**
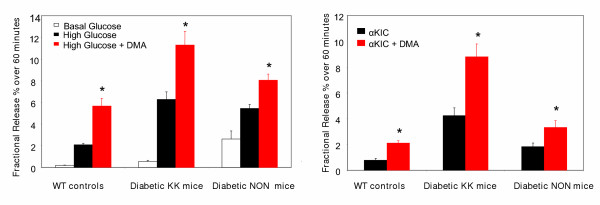
**DMA enhances insulin secretion stimulated by glucose and αKIC (alpha-ketoisocaproate), in islets isolated from both normal and diabetic mice: **Groups of islets were treated with different secretagogues with and without intracellular acidification produced by 40 μM DMA (denoted in red). Insulin secretion was measured at the end of the 60-minute stimulation period. Insulin secretion produced by both 16.7 mM glucose and 20 mM αKIC is significantly enhanced by intracellular acidification, in all three mouse strains (n = 10; * = p < 0.001). Values in the figure are expressed as fractional release (% of total insulin content released over the 60-minute period). The corresponding absolute amounts (ng/ml) for each condition from left to right are: 0.46 ± 0.1, 4.04 ± 0.44, 6.37 ± 0.71, 3.39 ± 0.72, 31.97 ± 2.58, 46.12 ± 5.51, 7.5 ± 0.9, 22.4 ± 3.2, 29.87 ± 5.35, 1.24 ± 0.18, 2.05 ± 0.3, 17.12 ± 2.11, 34.73 ± 4.053, 11.8 ± 1.38 and 13.76 ± 1.77.

DMA normally produces a dramatic increase in nutrient stimulated insulin secretion from isolated islets [7,8]. In this study we monitored the effect of DMA on the insulin response to glucose and αKIC in islets isolated from diabetic mice. DMA consistently enhanced insulin secretion in response to both secretagogues (glucose and αKIC), in islets from both diabetic models as well as from WT mice (Fig. 2).

**Figure 3:**
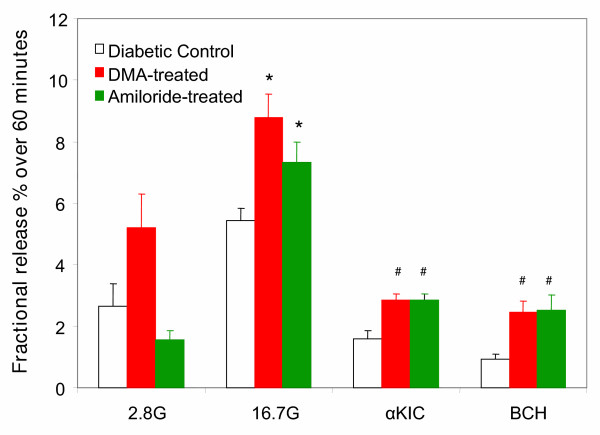
**Prior oral treatment with DMA and amiloride enhances insulin secretion in isolated islets from diabetic NON mice: **Islets were isolated from diabetic NON mice previously treated with DMA (estimated dose: 1 mg/kg/day) or amiloride (estimated dose: 5 mg/kg/day) in drinking water for a week. Groups of islets were treated with different secretagogues as indicated, and insulin secretion was measured at the end of the 60-minute stimulation period. Prior oral treatment with amiloride derivatives significantly enhances insulin secretion stimulated by each secretagogue. G = mM glucose; αKIC = alpha-ketoisocaproate (20 mM); BCH = 2-amino-bicyclo[2,2,1]heptane-2-carboxylic acid (20 mM); n = 5; * = p < 0.001 and # = p < 0.05, compared with each corresponding untreated control group. Values in the figure are expressed as fractional release (% of total insulin content released over the 60-minute period). The corresponding absolute amounts (ng/ml) for each condition from left to right are: 9.5 ± 1.29, 12.4 ± 3.06, 4.5 ± 1.05, 18.81 ± 2.65, 24.73 ± 1.96, 19.02 ± 3.02, 11.8 ± 1.38, 8.0 ± .66, 7.5 ± 0.96, 5.98 ± 1.48, 9.07 ± 2.1, and 8.92 ± 1.95

Since *in-vitro *treatment of isolated islets with DMA enhances NSIS, we next tested the corresponding effects of oral administration of DMA or amiloride to diabetic mice. Diabetic KK mice, being hyperinsulinemic, exhibited above-normal NSIS in the *in-vitro *experiments. Therefore, the *in-vivo *administration of amiloride was done only in the diabetic NON mice, which exhibited a significant impairment in NSIS. Diabetic NON mice were divided into three groups, i.e. DMA-treated; amiloride treated; and untreated control. DMA (1 mg/Kg/day) or amiloride (5 mg/Kg/day) was administered in drinking water to the test groups for one week prior to isolation of islets for insulin measurement. Mice were housed two to a cage and their behavior was monitored. All animals were observed to drink frequently, and drug dose was calculated using estimated water intake. Islets isolated from mice pre-treated with DMA or amiloride consistently exhibited a significantly stronger insulin response to all three secretagogues tested (16.7 mM glucose, 20 mM αKIC or BCH) (Fig. 3). Thus, oral treatment with low doses of DMA or amiloride enhances insulin release in islets from diabetic NON mice, with no conspicuous adverse effects on fluid or blood pressure regulation.

One problem we encountered with the diabetic NON mice was that the basal insulin release from isolated islets was high. Consequently the defect in NSIS was not obvious due to the elevated basal insulin release. However, the increase in insulin release produced by high glucose in NON mice (2 fold or less compared to basal glucose) is much smaller than that in the other two strains (10–12 fold compared to basal glucose), and non-glucose secretagogues such as αKIC and BCH do not stimulate above-basal insulin release in NON islets. Furthermore, the increased basal insulin release found in isolated islets may not directly translate into elevated plasma insulin, because the islet number per pancreas is diminished in NON mice, and the islets show abnormalities in size and shape as well. Thus, NON mice have a marked secretory defect, which is corrected to a significant degree by amiloride derivatives.

**Figure 4:**
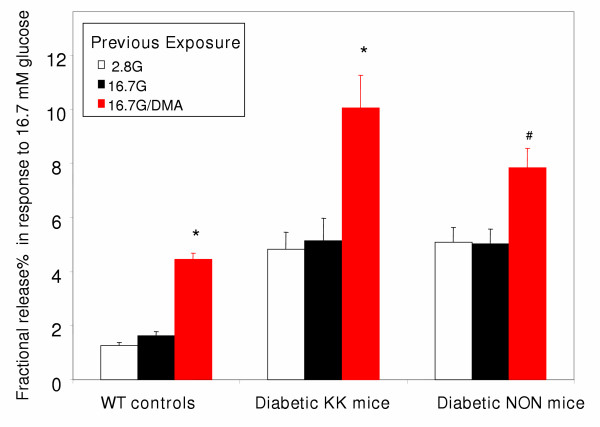
**DMA enables glucose to induce TDP in islets from both normal and diabetic mice: **Insulin secretion in response to high glucose (16.7 mM) is shown, in groups of islets *previously exposed *to glucose with and without intracellular acidification produced by 40 μM DMA (denoted in red). DMA treatment enables glucose to induce TDP both in wild type controls (left) and each strain of type 2 diabetes (Right). G = mM glucose; n = 5; * = p < 0.001 and # = p < 0.05, compared with each corresponding untreated control. Values in the figure are expressed as fractional release (% of total insulin content released over the final 40-minute period in response to 16.7 mM glucose). The corresponding absolute amounts (ng/ml) for each condition from left to right are: 2.88 ± 0.57, 5.3 ± 0.76, 8.5 ± 0.93, 11.96 ± 2.14, 20.32 ± 6.12, 39.68 ± 5.69, 15.2 ± 3.23, 15.79 ± 2.59, and 19.79 ± 3.41.

DMA treatment also unmasks TDP, a function normally absent in mouse islets [7]. TDP is defined as an enhancement of the insulin secretory response in the β cell, induced by a *previous *exposure to glucose or certain other secretagogues. TDP can be measured by comparing the glucose-induced insulin response in islets previously exposed high glucose or basal glucose. As shown in figure 4, treatment with DMA enables glucose to induce TDP in islets from both diabetic strains, albeit to a lesser degree than in WT islets.

These results show that islets isolated from diabetic mice exhibit some abnormalities in their pH_i_-regulation, and that several aspects of their insulin response are significantly improved by amiloride derivatives. Thus, treatment with low doses of amiloride derivatives shows promise in the therapy of type 2 diabetes. The next step is to determine whether these drugs can normalize blood glucose and insulin levels. These *in vivo *studies will be conducted in a more suitable model of type 2 diabetes with a more pronounced secretory defect, with parallel *in-vitro *work on human islets. Currently we are looking into suitable rodent models of NIDDM, and one mouse model that shows promise is the strain GK^lox/w ^+ Rip-Cre developed by another group at Vanderbilt [25]. The current study shows the potential value of amiloride in improving insulin release in diabetes, and recently-developed better amiloride analogs such as pyrazinoylguanidine [26,27] may be good candidates for correcting the secretory defect in human diabetes.

## Conclusion

This study demonstrates the presence of abnormalities in islet pH_i_-regulation in type 2 diabetes, and the ability of DMA and amiloride to significantly improve NSIS in isolated islets from diabetic mice. Thus, treatment with low doses of amiloride derivatives has potential therapeutic value for enhancing NSIS in human diabetes, and merits further investigation through *in vivo *studies.

## List of Abbreviations

NSIS: nutrient-stimulated insulin secretion; DMA: dimethyl amiloride; pH_i _= intracellular pH; TDP: time-dependent potentiation; KRBH: HEPES-buffered Krebs Ringer Bicarbonate solution; αKIC: alpha-ketoisocaproate; BCH: 2-amino-bicyclo[2,2,1]heptane-2-carboxylic acid; SNARF5-AM: carboxy-seminaphthorhodofluor-5 acetoxy methyl ester; SEM: standard error of mean; WT: wild type; Diabetic KK mice: KK/Upj-A<y>/J mice; Diabetic NON mice: NON/LtJ mice; MHC: Major histocompatibility complex.

## Competing interests

The author(s) declare that they have no competing interests.

## Authors' contributions

SCG designed the study and carried out the insulin secretion experiments, *in-vivo *treatments and intracellular pH-measurements. WSH prepared the cultured islets used in pH_i _measurements. DWP participated in the design and coordination of the study.

## Pre-publication history

The pre-publication history for this paper can be accessed here:



## References

[B1] Straub SG, Sharp GW (2002). Glucose-stimulated signaling pathways in biphasic insulin secretion. Diabetes Metab Res Rev.

[B2] Nesher R, Cerasi E (2002). Modeling phasic insulin release: immediate and time-dependent effects of glucose. Diabetes.

[B3] Henquin JC, Ishiyama N, Nenquin M, Ravier MA, Jonas JC (2002). Signals and pools underlying biphasic insulin secretion. Diabetes.

[B4] Nesher R, Cerasi E (1987). Biphasic insulin release as the expression of combined inhibitory and potentiating effects of glucose. Endocrinology.

[B5] Cerasi E (1975). Potentiation of insulin release by glucose in man. II. Role of the insulin response, and enhancement of stimuli other than glucose. Acta Endocrinologica.

[B6] Grill V, Rundfeldt M (1979). Effects of priming with D-glucose on insulin secretion from rat pancreatic islets: Increased responsiveness to other secretagogues. Endocrinology.

[B7] Gunawardana SC, Rocheleau JV, Head WS, Piston DW (2004). Nutrient-stimulated insulin secretion in mouse islets is critically dependent on intracellular pH. BMC Endocr Disord.

[B8] Gunawardana SC, Sharp GW (2002). Intracellular pH plays a critical role in glucose-induced time-dependent potentiation of insulin release in rat islets. Diabetes.

[B9] Pace CS (1984). Role of pH as a transduction device in triggering electrical and secretory responses in islet B cells. Federation Proceedings.

[B10] Smith JS, Pace CS (1983). Modification of glucose-induced insulin release by alteration of pH. Diabetes.

[B11] Pace CS, Tarvin JT, Smith JS (1983). Stimulus-secretion coupling in beta-cells: modulation by pH. Am J Physiol.

[B12] Best L, Bone EA, Meats JE, Tomlinson S (1988). Is intracellular pH a coupling factor in nutrient-stimulated pancreatic islets?. J Mol Endocrinol.

[B13] Best L, Yates AP, Gordon C, Tomlinson S (1988). Modulation by cytosolic pH of calcium and rubidium fluxes in rat pancreatic islets. Biochem Pharmacol.

[B14] Best L, Elliot AC (1995). Changes in 2',7'-bis(carboxyethyl) 5'(6')-carboxyflurescein, fura-2 and autofluorescence in intact rat pancreatic islets in response to nutrients and non-nutrients. Mol Cel Endocrinol.

[B15] Sener A, Hutton JC, Kawazu S, Boschero AC, Somers G, Devis G, Herchuelz A, Malaisse WJ (1978). The stimulus-secretion coupling of glucose-induced insulin release; Metabolic and functional effects of NH_4_^+ ^in rat islets. J Clin Invest.

[B16] Lebrun P, Van Ganse E, Juvent M, Deleers M, Herchelz A (1982). Na^+^-H^+ ^exchange in the process of glucose-induced insulin release from the pancreatic B-cell. Effects of amiloride on 86Rb, 45Ca fluxes and insulin release. Biochim Biophys Acta.

[B17] Lynch AM, Meats JE, Best L, Tomlinson S (1989). Effects of nutrient and non-nutrient stimuli on cytosolic pH in cultured insulinoma (HIT-T15) cells. Biochim Biophys Acta.

[B18] Lacy PE, Kostianovsky M (1967). Method for the isolation of intact islets of Langerhans from the rat pancreas. Diabetes.

[B19] Arkhammar POG, Terry BR, Kofod H, Thastrup O (1998). Pancreatic islets cultured on extracellular matrix: An excellent preparation for microfluorometry. Methods Cell Sci.

[B20] Rocheleau JV, Head WS, Nicholson WE, Powers AC, Piston DW (2002). Pancreatic islet beta-cells transiently metabolize pyruvate. J Biol Chem.

[B21] Patterson GH, Knobel SM, Arkhammar P, Thastrup O, Piston DW (2000). Separation of the glucose-stimulated cytoplasmic and mitochondrial NAD(P)H responses in pancreatic islet beta cells. Proc Natl Acad Sci USA.

[B22] Liu J, Diwu Z, Leung WY (2001). Synthesis and photophysical properties of new fluorinated benzo(*c*)xanthene dyes as intracellular pH indicators. Bioorg Med Chem Let.

[B23] JAX^R ^Mice Data Sheet: Jackson Laboratory Database. http://jaxmice.jax.org/jaxmice-cgi/jaxmicedb.cgi?objtype=pricedetail&stock=002468&dest=N.

[B24] JAX^R ^Mice Data Sheet: Jackson Laboratory Database. http://jaxmice.jax.org/jaxmice-cgi/jaxmicedb.cgi?objtype=pricedetail&stock=002423&dest=N.

[B25] Postic C, Shiota M, Niswender KD, Jetton TL, Chen Y, Moates JM, Shelton KD, Lindner J, Cherrington AD, Magnuson MA (1999). Dual roles for glucokinase in glucose homeostasis as determined by liver and pancreatic beta cell-specific gene knock-outs using Cre recombinase. J Biol Chem.

[B26] Vesell ES, Beyer KH (2000). Studies on pyrazinoylguanidine: a novel antihypertensive, hypoglycemic and lipolytic drug intended for adjunctive use in hypertensive patients with type 2 diabetes mellitus. Toxicolog.

[B27] Vesell ES, Beyer KH (1999). Studies on pyrazinoylguanidine. 7. Effects of single oral doses in normal human subjects. Pharmacology.

